# Diurnal Changes in Active Carbon and Nitrogen Pathways Along the Temperature Gradient in Porcelana Hot Spring Microbial Mat

**DOI:** 10.3389/fmicb.2018.02353

**Published:** 2018-10-02

**Authors:** María E. Alcamán-Arias, Carlos Pedrós-Alió, Javier Tamames, Camila Fernández, Danilo Pérez-Pantoja, Mónica Vásquez, Beatriz Díez

**Affiliations:** ^1^Department of Oceanography, Universidad de Concepción, Concepción, Chile; ^2^Department of Molecular Genetics and Microbiology, Pontificia Universidad Católica de Chile, Santiago, Chile; ^3^Center for Climate and Resilience Research, Universidad de Chile, Santiago, Chile; ^4^Programa de Biología de Sistemas, Centro Nacional de Biotecnología – Consejo Superior de Investigaciones Científicas, Madrid, Spain; ^5^Laboratoire d’Océanographie Microbienne, Observatoire Océanologique, Sorbonne Universités, Université Pierre-et-Marie-Curie, Centre National de la Recherche Scientifique, Banyuls-sur-Mer, France; ^6^Fondap IDEAL, Universidad Austral de Chile, Valdivia, Chile; ^7^Programa Institucional de Fomento a la Investigación, Desarrollo e Innovación, Universidad Tecnológica Metropolitana, Santiago, Chile

**Keywords:** *Cyanobacteria*, carbon and nitrogen assimilation, neutral hot spring, metagenomics, metatranscriptomics, microbial mat, photosynthesis

## Abstract

Composition, carbon and nitrogen uptake, and gene transcription of microbial mat communities in Porcelana neutral hot spring (Northern Chilean Patagonia) were analyzed using metagenomics, metatranscriptomics and isotopically labeled carbon (H^13^CO_3_) and nitrogen (^15^NH_4_Cl and K^15^NO_3_) assimilation rates. The microbial mat community included 31 phyla, of which only *Cyanobacteria* and *Chloroflexi* were dominant. At 58°C both phyla co-occurred, with similar contributions in relative abundances in metagenomes and total transcriptional activity. At 66°C, filamentous anoxygenic phototrophic *Chloroflexi* were >90% responsible for the total transcriptional activity recovered, while *Cyanobacteria* contributed most metagenomics and metatranscriptomics reads at 48°C. According to such reads, phototrophy was carried out both through oxygenic photosynthesis by *Cyanobacteria* (mostly *Mastigocladus*) and anoxygenic phototrophy due mainly to *Chloroflexi*. Inorganic carbon assimilation through the Calvin–Benson cycle was almost exclusively due to *Mastigocladus*, which was the main primary producer at lower temperatures. Two other CO_2_ fixation pathways were active at certain times and temperatures as indicated by transcripts: 3-hydroxypropionate (3-HP) bi-cycle due to *Chloroflexi* and 3-hydroxypropionate-4-hydroxybutyrate (HH) cycle carried out by *Thaumarchaeota*. The active transcription of the genes involved in these C-fixation pathways correlated with high *in situ* determined carbon fixation rates. *In situ* measurements of ammonia assimilation and nitrogen fixation (exclusively attributed to *Cyanobacteria* and mostly to *Mastigocladus* sp.) showed these were the most important nitrogen acquisition pathways at 58 and 48°C. At 66°C ammonia oxidation genes were actively transcribed (mostly due to *Thaumarchaeota*). Reads indicated that denitrification was present as a nitrogen sink at all temperatures and that dissimilatory nitrate reduction to ammonia (DNRA) contributed very little. The combination of metagenomic and metatranscriptomic analysis with *in situ* assimilation rates, allowed the reconstruction of day and night carbon and nitrogen assimilation pathways together with the contribution of keystone microorganisms in this natural hot spring microbial mat.

## Introduction

Microbial mats have been studied for decades as model systems for testing principles of microbial ecology (Stal, 1995; Ward, 2006; Hou et al., 2013; Inskeep et al., 2013). Microbial mats in hot springs are dynamic and relatively simple ecosystems exhibiting spatial and temporal heterogeneity (Ward et al., 1998; Ward, 2006; Bhaya et al., 2007; Klatt et al., 2011; Alcamán et al., 2015). These microenvironments support a diversity of species carrying out a wide range of metabolic processes (Jensen et al., 2011; Kim et al., 2015). The upper few millimeters in alkaline and neutral mats are dominated by oxygenic phototrophic cyanobacteria, such as the unicellular cyanobacterium *Synechococcus* spp. (Steunou et al., 2006, 2008; Bhaya et al., 2007; Jensen et al., 2011; Klatt et al., 2011), the filamentous non-heterocystous *Oscillatoria* spp. and the filamentous heterocystous *Mastigocladus* spp. (Stewart, 1970; Miller et al., 2006; Mackenzie et al., 2013; Alcamán et al., 2015), as well as the filamentous anoxygenic phototrophs (FAPs) *Roseiflexus* sp. and *Chloroflexus* sp. (van der Meer et al., 2010; Klatt et al., 2011; Liu et al., 2011; Kim et al., 2015). Several diversity studies in hot spring microbial mats have shown that members of the phototrophic *Cyanobacteria* and *Chloroflexi* phyla can co-exist, sometimes in a collaborative manner (Liu et al., 2011). For instance, *Synechococcus* sp. produces low-molecular weight organic compounds as by-products of its metabolism (as primary producers), that are assimilated photoheterotrophically by FAPs (Sandbeck and Ward, 1981; Anderson et al., 1987; Bateson and Ward, 1988).

During daytime light penetrates only a few millimeters into the microbial mat (Kühl and Jørgensen, 1992), and the upper part of the mat is supersaturated with oxygen photosynthetically produced by *Cyanobacteria* (Canfield and Des Marais, 1994; Jensen et al., 2011) but the concentration of oxygen rapidly diminishes with depth. On the other hand, anoxygenic photosynthetic microorganisms, such as *Chloroflexi*, may use sulfide (Madigan and Brock, 1977) or hydrogen (Holo and Sirevåg, 1986) as electron donors and fix CO_2_, through the 3-hydroxypropionate (3-HP) bi-cycle (Strauss and Fuchs, 1993; Klatt et al., 2007; Zarzycki et al., 2009; Zarzycki and Fuchs, 2011). Moreover, *Chloroflexi* can simultaneously incorporate inorganic (CO_2_) and organic carbon such as acetate (generated under anoxic conditions at night) or glycolate (generated by photorespiration under O_2_ supersaturation during the day) produced by *Cyanobacteria* (Bateson and Ward, 1988; van der Meer et al., 2005, 2007; Zarzycki and Fuchs, 2011; Bryant et al., 2012). Therefore, the availability and abundance of these inorganic and organic compounds are factors that shape the relative degree to which FAPs behave as heterotrophs, mixotrophs or autotrophs (van der Meer et al., 2005; Zarzycki and Fuchs, 2011; Klatt et al., 2013a; Urschel et al., 2015). However, the dynamics on a daily basis and along the temperature gradient of the different autotrophic strategies for inorganic carbon fixation by thermophiles are still poorly characterized.

The nitrogen cycle also shows a variety of potentially active pathways in thermal mats, such as N_2_-fixation attributed to *Cyanobacteria* (such as *Synechococcus* sp. and *Mastigocladus* sp.) (Miller et al., 2006; Steunou et al., 2006, 2008; Alcamán et al., 2015), ammonia oxidation by Archaea (Candidatus *Nitrosocaldus yellowstonii*) (de la Torre et al., 2008; Reigstad et al., 2008; Hamilton et al., 2014; Thiel et al., 2016), and denitrification and dissimilatory nitrate reduction to ammonia (DNRA) attributed to *Aquificales* and *Thermales* (*Hydrogenobacter* sp., *Sulfurihydrogenibium* sp., *Anoxybacillus* sp. and *Thermus* sp.) (Dodsworth et al., 2011b). Hot springs are commonly N-limited systems due to the fast assimilation and turnover of inorganic nitrogen forms (Alcamán et al., 2015; Lin et al., 2015). Thus, N_2_-fixation carried out by *Cyanobacteria* has been reported to be the most relevant biological process for the input of exogenous nitrogen to microbial mats in Porcelana (Alcamán et al., 2015). Evidence of a balance between inputs and outputs of N was demonstrated by Hamilton et al. (2014) in Yellowstone National Park (YNP) Perpetual Spouter hot spring (pH 7.03, 86.4°C), where a rapid depletion of bioavailable nitrogen by putatively oxidizing *Thaumarchaeota* lead to an increase in the activity of the putative nitrogen-fixing bacterium *Thermocrinis albus* (phylum *Aquificae*), in the absence of other nitrogen sources. It has also been reported that Archaea are able to carry out different N reductive pathways, including nitrate assimilation, N_2_ fixation, and dissimilatory reactions (Cabello et al., 2004).

Recent development of high-throughput sequencing techniques has expanded knowledge of taxonomical diversity in hot springs (Bhaya et al., 2007; Klatt et al., 2011; Liu et al., 2011, 2012b; Thiel et al., 2016). Metagenomics and metatranscriptomics are now most effective approaches to target the community structure and the expressed genes to reveal community functions carried out at a specific time (Bhaya et al., 2007; Urich et al., 2008; Liu et al., 2011; Klatt et al., 2013b; Thiel et al., 2016). Some metagenomics studies have revealed new aspects of microbial diversity distribution with temperature in hot springs (Bhaya et al., 2007; Klatt et al., 2013b), as well as the characterization of dominant phototrophic populations and previously unidentified members of the *Chloroflexi* phylum (Klatt et al., 2011). Also, putative ecotypes adapted to different niches have been found within the undermat, particularly of *Roseiflexus* spp. (Thiel et al., 2016). In addition, metatranscriptomics has provided important information about temporal patterns of key gene transcription in processes such as N_2_-fixation (*nif*H), oxygenic (*psa*A) and anoxygenic (*puf*M) photosynthesis (Liu et al., 2011), as well as survival strategies of relevant populations in hot springs (Quaiser et al., 2014).

Most metagenomics and metatranscriptomics studies, however, have concentrated on just one spot per spring or just one diel cycle at a particular point ignoring changes along the temperature gradient (Liu et al., 2011; Klatt et al., 2013a,b; Thiel et al., 2017). Thus, the activities and corresponding gene transcription patterns of all such autotrophic carbon and nitrogen pathways, and the organisms responsible for each one of them, are not well understood along temperature gradients and at different times. In the present study, we used metagenomics, metatranscriptomics, and biogeochemical analyses (isotopic assimilation rates) to clarify the structure and activities of the microbial mat communities along a moderately thermophilic gradient (66, 58, and 48°C) at noon and night in the neutral hot spring of Porcelana (Northern Patagonia, Chile). Within an area of rainforest, the water surfaces close to 70°C, runs downstream to join River Porcelana, cooling down to approximately 40°C in its transit. The pH is close to neutral and the channel walls are heavily colonized by microbial mats, first orange and later green in color. Porcelana hot spring had only received some sporadic visits determining a few physicochemical parameters (Hauser, 1989; Waring, 1965), but no microbiological studies had been carried out until 2013. We analyzed the taxonomic composition of the mats by DGGE and sequencing (Mackenzie et al., 2013) and measured nitrogen fixation in a diel study attributing all the activity to the cyanobacteria *Mastigocladus* sp. (Alcamán et al., 2015).

## Materials and Methods

### Sampling

**FIGURE 1:**
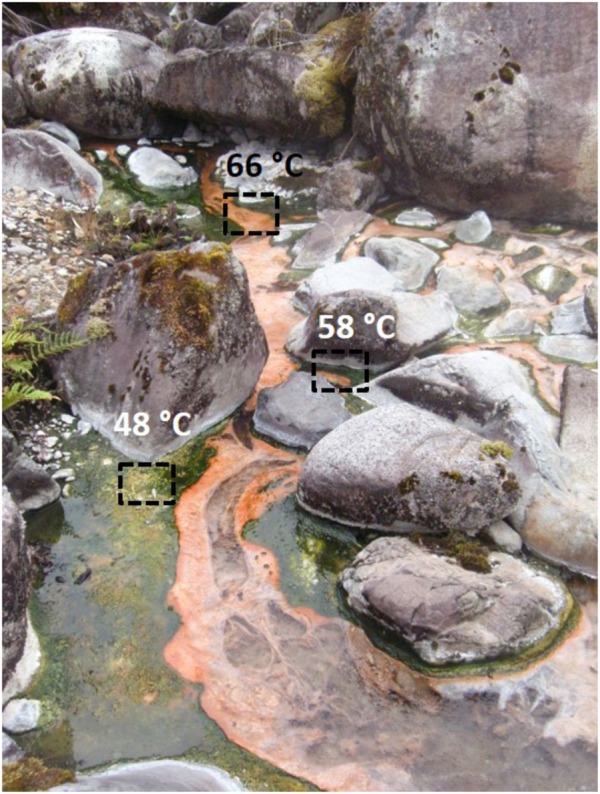
Porcelana hot spring and the pigmented microbial mats growing along the temperature gradient. Sampling sites are indicated by squares: 66°C (orange), 58°C (orange + green) and 48°C (green).

Porcelana hot spring is located in Chilean Patagonia (42° 27′ 29.1″S – 72° 27′ 39.3″W) at the end of the Comau Fjord and is part of an area of shallow depth geothermic events whose outflow pours over volcanic rocks originated in the Quaternary Period and is encircled by active Quaternary volcanoes such as the Huequi Volcano (Duhart et al., 2000). It is a slightly acidic pH (∼6.5) system with a maximal temperature of 66°C when was sampled in March 2013 (**Figure 1**). The channel walls are heavily colonized by microbial mats, first orange and later green in color. Microbial mats (up to 2 cm thick) grow from 70 to 46°C downstream, representing a stable biogeochemical system as previously reported by Mackenzie et al. (2013) and Alcamán et al. (2015). Colorful microbial mats growing at 66 (orange mat), 58 (orange and green mat) and 48°C (green mat) (**Figure 1**), were sampled using a cork borer with a diameter of 7 mm. Cores 1 cm thick were collected in triplicate at noon (12:00 h), and also at night (23:00 h) at 66, 58, and 48°C. Isotopic nitrogen (^15^NH_4_Cl, and K^15^NO_3_) and carbon (H^13^CO_3_) uptake experiments were performed in microbial mats growing at 58 and 48°C (but not at 66°C), cores were collected in triplicate as explained above. Samples for DNA and RNA were transported in liquid nitrogen and kept at -80°C until analysis. In the laboratory, DNA and RNA for sequencing analyses (metagenomics/metatranscriptomics) were extracted from pooled triplicate and homogenized mat samples.

### Stable Isotope Nitrogen and Carbon Uptake Experiments

Incubations at 58 and 48°C were run *in situ* under light and dark conditions. Unfortunately, assays at 66°C were not possible due to logistic problems at the time of sampling. For each sample point, three microbial mat cores (7 mm in diameter and 1 cm thick) were mixed to homogenize potential spatial heterogeneity and transferred to pre-sterilized 10 ml vials with 1 ml of pre-filtered (0.2 μm filter pore) spring water from the same sampling point. Dual ^15^N/^13^C uptake experiments were conducted independently by adding 50 μL of ^15^NH_4_Cl (500 μmol L^-1^) plus 500 μl of H^13^CO_3_ (500 μmol L^-1^), and 30 μl K^15^NO_3_ (500 μmol L^-1^) plus 500 μL of H^13^CO_3_ (500 μmol L^-1^) in parallel. These incubations were run in triplicate for biological replication. The ^15^N tracer additions were generally kept close to 10% of natural abundance of nitrate (which was 3.2 μmol L^-1^ NO_3-_) and ammonium (natural abundance was 0.04 μmol L^-1^ NH_4+_). The nitrogen fixation (^15^N_2_) experiments were carried out simultaneously but have been published separately (see details in Alcamán et al., 2015). Briefly, 1 ml of ^15^N_2_ gas (98% atom ^15^N_2_ gas; Sigma-Aldrich) was added through a gas-tight syringe into the headspace of each vial. For each temperature analyzed, two replicates without the isotope (^15^N and ^13^C) were also incubated in order to determine the natural isotopic composition and to be used as negative controls. Vials were incubated at 58 and 48°C *in situ* for 6 h. After incubations, mat samples were dried at 70°C for 48 h. Isotopic analyses of ^15^N and ^13^C were carried out using an IRMS delta plus Thermo FinniganH Mass Spectrometer (Stable Isotope Laboratory, Granada, Spain). Rates of carbon fixation and nitrogen assimilation were expressed as daily carbon or nitrogen assimilation rates (nmoles cm^-2^ d^-1^), and calculated as:

(1) $$\begin{array}{c} \%R_{\mathrm{exc}}=\frac{\left(\frac{V_{\mathrm{add}}*T_{\mathrm{conc}}}{V_{\mathrm{inc}}}\right)+Nat_{\mathrm{conc}}*Nat_{\mathrm{abundance}}}{Nat_{\mathrm{conc}}+\frac{V_{\mathrm{add}}*T_{\mathrm{conc}}}{V_{\mathrm{inc}}}}\;*100-(\%)Nat_{\mathrm{abundance}} \end{array}$$

(2) $$\begin{array}{c} \rho =\left[\frac{\left(\%AT_{f}-Nat_{\mathrm{abundance}}\right)}{\%R_{\mathrm{exc}}}\right]*[PON\;or\;POC]/mg/Time_{\mathrm{inc}} \end{array}$$

where %*R*_exc_, the excess enrichment of the tracer after inoculation, is calculated using Eq. (1): *V*_add_ (L^-1^) indicates the volume of isotope added to the sample; *T*_conc_ (mol L^-1^) is the tracer concentration added to the sample; *V*_inc_ (L^-1^) is the sample volume incubated; Nat_conc_ (mol L^-1^) represents the initial amount C or N in the sample after isotope added; Nat_abundance_ (mol L^-1^) corresponds to natural abundance of C or N in the mat. A fixed dissolved inorganic carbon value of 0.8 mM was used based on measurements in the Porcelana mat (Alcamán et al., 2015). The assimilation rate (ρ) was calculated with Eq. (2), where %AT_f_ is the percentage of total atoms after incubation; PON or POC (mg) are the amounts of particulate organic nitrogen or carbon recovered after incubation and measured by mass spectrometry; mg is the microbial mat mass (dry weight) analyzed in mass spectrometry; and Time_inc_ is the incubation time (h). The final rates were extrapolated per core area (cm^-2^) and as daily rate (d^-1^).

Turnover time of ammonium and nitrate, at the time of sampling was estimated using the uptake rate obtained by dividing the *in situ* ammonium and nitrate natural concentrations by their respective uptake rates, giving units of hours. Concentrations in water did not change over 24 h in our previous studies (i.e., Alcamán et al., 2015).

### Nucleic Acid Extraction and High Throughput Sequencing

The whole contents of each previously homogenized mat sample were extracted as previously described in Alcamán et al. (2015). Briefly for DNA, glass beads were added to the sample, which then was homogenized by bead beating three times for 20 s, followed by an organic Phenol–Chloroform extraction. Usually between 0.4 and 0.7 g of mat-samples were used. In the case of RNA samples, the Trizol-mat mixture was subjected to beating only twice. Quality and quantity of the extracted nucleic acids were checked by spectrophotometer (NanoDrop Technologies Inc., Wilmington, DE, United States) and by 0.8% agarose gel DNAse/RNAse-free, and the material was kept at -80°C. Then just one DNA (∼600 ng) and one RNA (1 μg) sample was sent to sequencing for each temperature and time of day using Illumina technology (Research and Testing Laboratory, Lubbock, TX, United States). The metagenomes and metatranscriptomes were sequenced. Briefly, enzymatic fragmentation was done prior to DNA library construction using NEBNext dsFragmentase, then fragmented DNA was cleaned up by column purification. The construction of libraries (Ultra DNA Library Prep Kit for Illumina) was conducted by: End prep, adaptor ligation, size selection of adaptor-ligated DNA, PCR amplification and product purification. Finally, fragment size was checked by Fragment Analyzer (Advanced Analytical Technologies, Ankeny, IA, United States). For metatranscriptomes, total RNA (1 μg) was cleaned up of rRNA prior to library construction by Ribo-Zero rRNA Removal Kit Bacteria (Illumina, San Diego, CA, United States) according the manufacturer’s instruction followed by clean up using Agencourt RNAClean XP Kit (Beckman Coulter, Indianapolis, IN, United States). A gel-free or low input small RNA library prep kit with reduced bias for Illumina sequencing was used. The specific kit (NEXTflex^TM^ Illumina Small RNA Sequencing Kit v3) and all previous mentioned protocols are available at http://www.biooscientific.com/next-gen-sequencing/nextflex-illumina-small-rna-seq-library-prep-kits (Bioo Scientific, Austin, TX, United States). Unfortunately, it was not possible to obtain a metatranscriptome of sufficient quality for the 66°C sample collected at night. All read and transcript sequences, corresponding to metagenomes and metatranscriptomes, obtained from this study have been deposited in the Sequence Read Archive under the accession number SRP104009.

### Taxonomic Assignments of Reads

To ensure feasibility of downstream analysis, FastQC^1^ was used to assess the quality of the sequence data. To correct the quality issues found Cutadapt^2^ was used, leaving only mappable sequences longer than 30 bp (-m 30), with a 3′ end trimming for bases with quality below 28 (-q 28), a hard clipping of the first five leftmost bases (-u 5), and finally a perfect match of at least 10 bp (-O 10) against the standard Illumina adaptors. This procedure reduced the total number of sequences from 452.7 to 394.7 million (**Supplementary Table S1**).

For the classification of 16S rRNA gene sequences, the rRNA reads in DNA and RNA high quality samples were identified and separated using Ribopicker (Schmieder et al., 2012) with the non-redundant rRNA database that combines Silva, Greengenes, RDP-II, NCBI archaeal/bacterial, HMP and Rfam databases. Ribosomal RNA sequences represented 4.2% of the total, with 378.2 million of reads related to functional genes (**Supplementary Table S1**). The BLAST parameters used were: e-value 1e^-10^, min-score 200 and min query coverage of 0.95.

Taxonomical assignment of the non-rRNA high quality reads was conducted by Diamond (Buchfink et al., 2015) with default parameters and by the NCBI non-redundant (NR) database, previously filtered by bacterial sequences (ids obtained from gbbct database^3^) and added with new bacterial genomes not incorporated yet from JGI^4^. The NCBI taxonomic identifiers (taxid) for each match were manually added as a new column in Diamond’s output (csv file), using an in-house bash script based on Unix command line programs that take each GI code and match it with the corresponding taxid from the NCBI taxonomy tree (Sayers et al., 2009). Subsequently, the results were parsed using the lowest common ancestor algorithm trough MEGAN 5 (Huson et al., 2007) under default parameters (score = 50). The total number of non-redundant hits was 114.2 million. On average around 86.6% of the reads could be taxonomically assigned in most samples. Only in the 66°C cDNA day sample, the percentage was near 20% (**Supplementary Table S1**).

Taxonomic assignment of reads is a process subject to biases and errors. This is particularly the case when the organisms studied have few reference genomes in databases. In these cases, taxonomic assignment will tend to assign reads to genomes in the database, even if the match is not very good. Thus, we also used the rDNA sequences extracted with riboPicker for taxonomic assignment of the most relevant organisms in the mat, both in metagenomes and metatranscriptomes. In this case, other problems appear, for example the difference in copy number among different microorganisms will alter the relative abundances in an unknown way. For the present analysis, we will use the metagenomic assignments (**Supplementary Table S2**), because our interest is to analyze them together with metatranscriptomes, which will logically have similar biases to metagenomes. However, we will use the 16S rDNA sequences to confirm the identity of the main microorganisms discussed (**Supplementary Table S3**), accession numbers SUB4537948.

### Co-assembly of Metagenomes and Metatranscriptomic Functional Assignments

To identify the specific pathways of photosynthesis, CO_2_ fixation and nitrogen metabolisms, the three daytime metagenomes (66, 58, and 48°C) were co-assembled using the Spades software (Bankevich et al., 2012). Key genes from pathways of interest were used as indicators of the importance of each pathway (**Supplementary Table S4**). This was done using the normalized abundance of reads corresponding to each relevant gene in either metagenomes or metatranscriptomes. The raw counts were normalized to fragments per kilobase and million reads using a custom script. Prodigal software (Hyatt et al., 2010) was used to predict genes for taxonomic and functional annotations. A homology search with the amino acid translations of the genes was done using RapSearch2 (Zhao et al., 2012) against the GenBank non-redundant protein database. Functional annotation in KEGG and COG codes was done using the fun3 software, as described in Guazzaroni et al. (2013). The abundance of genes in each sample was determined by mapping the corresponding reads to the co-assembled metagenomic contigs. We used Bowtie2 (Langmead and Salzberg, 2012) for that purpose, and quantified the number of mapped reads to each gene using HTSeq (Anders et al., 2015). The raw counts were normalized to fragments per kilobase and million reads using a custom script. Metatranscriptomic reads were also mapped against metagenomic contigs to obtain the abundance of transcripts for each gene. The mapping was conducted with the BBMap software using a minimum identity of 95%, *k* = 9, and other default parameters (Bushnell, 2014). We assumed that the transcripts that did not map to the reference contigs of the metagenome correspond to highly expressed genes of rare species that could not be assembled because of their low abundance in the metagenome. To include these genes in the analysis, we extracted the unmapped transcripts from the three metatranscriptomes and assembled them using Spades. The resulting new contigs from the metatranscriptomes (composed often by a single gene, but also of polycistrons corresponding to operons) were added to the metatranscriptomics contig set and treated as described above (taxonomic and functional assignment, mapping and quantification of transcripts).

## Results and Discussion

### DNA and cDNA Taxonomic Assignments Reveal Cyanobacteria and Chloroflexi as Main Players in the Mat

**FIGURE 2:**
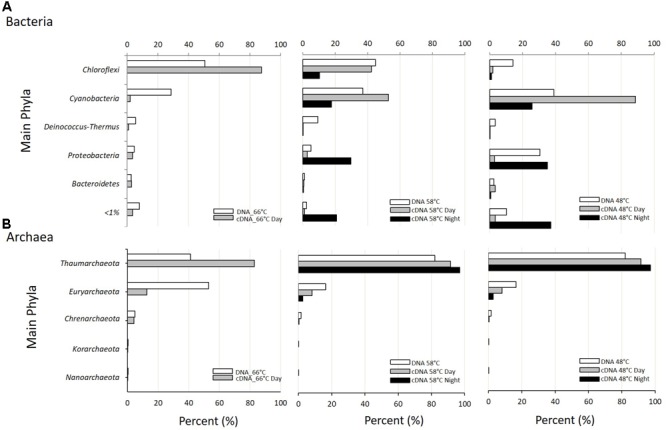
Taxonomic assignment at the Phylum level of metagenomic (DNA; white bars) and metatranscriptomic reads (cDNA day: gray bars; cDNA night: black bars) from samples at the three temperatures studied. **(A)** Percent of Bacteria reads assigned to the most abundant Phyla. **(B)** Percent of Archaea reads assigned to the most abundant Phyla.

The same 36 phyla (31 bacterial and 5 archaeal) were present at the three temperatures sampled regardless of time of day (**Supplementary Table S2**). Two bacterial phyla, *Cyanobacteria* (green mat) and *Chloroflexi* (orange mat), accounted for most of the reads in both metagenomes and metatranscriptomes (**Figure 2A**). *Chloroflexi* showed higher relative number of reads in metagenomes and metatranscriptomes at 66°C, decreasing at 48°C. On the contrary, *Cyanobacteria* reached their maximal abundance and activity (up to 80% of all reads) at 48°C at daytime. At the intermediate temperature of 58°C, *Chloroflexi* and *Cyanobacteria* were represented in similar abundance both in metagenomic and day and night metatranscriptomic libraries (**Figure 2A**). Notoriously, *Proteobacteria* showed their highest transcriptional activity (>50%) at 58 and 48°C during periods of darkness, unlike *Cyanobacteria* and *Chloroflexi* that showed more transcripts during daytime (**Figure 2A**).

Most filamentous anoxygenic bacteria (FAPs) of the phylum *Chloroflexi*, belonged to the order *Chloroflexales* (**Supplementary Figure S1a**). Members of Order *Chloroflexales* dominated at all temperatures and time of day studied and contributed with more than 90% to the total diurnal transcriptional activity. Most *Chloroflexales* reads belonged to two genera: *Chloroflexus* and *Roseiflexus*. The 16S rDNA sequences retrieved showed 96.9% and 98.9% similarity to *Chloroflexus aurantiacus* J-10-fl and *Roseiflexus* sp. RS-1, respectively (**Supplementary Table S3**).

*Oscillochloris trichoides* (99.6% similarity with strain DG-6 OSCT) was also present but in very low abundance (**Supplementary Figure S1b**). The novel member of *Chloroflexi, Ca.* Chloranaerofilum corporosum found in Mushroom Spring (Thiel et al., 2016), was specifically searched for in our metagenomes but we did not find it.

*Chloroflexus* sp. showed maximal abundance and transcriptional activity at 58°C, but transcriptional activity was also high at 66°C representing more than 70% of the transcriptional activity at this temperature. *Roseiflexus* sp., in turn, had maximal abundance at 66°C but represented ≤30% of the *Chloroflexi* transcriptional activity, whereas its transcriptional activity was highest (>90%) at 48°C at both day and night, compared to *Chloroflexus* (**Supplementary Figure S1b**). *Roseiflexus* sequences were less abundant at the intermediate temperature of 58°C in the metagenomes and metatranscriptomes. This might be due to different strains adapted to different temperature ranges. In effect, Nübel et al. (2002) found three *Roseiflexus*-like clades with temperature optima at 60 to 70°C, 47 to 53°C, 35 to 41°C. Similarly, our results indicate the presence of two populations with different temperature optima.

Klatt et al. (2011) mentioned an *Anaerolinea*-like chlorophototroph, later named *Ca*. Roseilinea gracile (Tank et al., 2017) in microbial mats in YNP. However, the sequences found in Porcelana were closer to *Anaerolinea thermophila* (NC_014960.1) than to *Ca*. Roseilinea gracile (**Supplementary Table S3**). To double check the presence of *Roseilinea*, its genome was used to recruit sequences from our metagenomes (with 95% minimal coverage) finding only around 490 sequences (with 95–100% identity). Therefore, *Roseilinea* would be found in barely detectable amounts (less than 0.001% of the abundance). In any case, the *Anaerolinea* sequences represented a very minor fraction of both metagenomes and metatranscriptomes (**Supplementary Figure S1**).

The phylum *Cyanobacteria* was represented by unicellular members of subsection II (Order *Chroococcales*), filamentous non-heterocystous members of subsection III (Order *Oscillatoriales*), and by filamentous heterocystous members of subsections IV (Order *Nostocales*) and V (Order *Stigonematales*) (**Supplementary Figure S2a**). Apart from the few reads of *Oscillatoriaceae* found at higher temperatures, most cyanobacterial reads corresponded to the single genus *Mastigocladus* (Order *Stigonematales*), a group of branching filamentous heterocystous cyanobacteria. This was confirmed by looking at the 16S rRNA genes retrieved from the metagenomes that showed 99.9% similarity to *Fischerella* sp. NIES-3754 (**Supplementary Table S3**). Dominance by this cyanobacterium has also been found in many hot springs around the world (Finsinger et al., 2008; Kaštovský and Johansen, 2008; McGregor and Rasmussen, 2008; Lukavsky et al., 2011), although not always in such high abundance (Miller et al., 2007; McGregor and Rasmussen, 2008) as in Porcelana. At the lowest temperatures, the *Cyanobacteria* contributed most of the total transcripts detected (>90%) during the day (**Figure 2A**), due mostly to the *Stigonematales* that, in turn, were largely (>90%) represented by members of the genus *Mastigocladus* sp. (**Supplementary Figure S2b**). *Nostocales* were also abundant (<40%) but with low (<10%) representation in metatranscriptomes at 48°C (**Supplementary Figure S2a**). At 66°C, *Oscillatoriales* were the most transcriptionally active members of cyanobacteria (>75%) (**Supplementary Figure S2a**). It must be remembered, however, that cyanobacteria at this temperature were a smaller percent of the total reads and, thus, most of the cyanobacterial abundance and transcriptional activity were those of *Mastigocladus* at the two lower temperatures (**Supplementary Figure S2b**). During the night, *Oscillatoriales* and *Chroococcales* contributed in a similar level to the cyanobacterial transcriptional activity at 58°C. At 48°C, in turn *Chroococcales* and *Stigonematales* were the most active (based on normalized abundance of reads in metatranscriptomes, **Supplementary Figure S2a**).

Dominance by *Cyanobacteria* and FAPs is common in most hot spring mats at the range of temperatures covered here. For example, in the slightly alkaline Mushroom and Octopus springs in YNP, *Cyanobacteria* and *Chloroflexi* dominated the temperature range between 30 and 70°C (Miller et al., 2009; Liu et al., 2011; Klatt et al., 2013a; Thiel et al., 2016). Whereas in Porcelana and YNP the same main *Chloroflexi* (*Chloroflexus* and *Roseiflexus*) were recovered, *Cyanobacteria* were rather different. In YNP, *Synechococcus* spp. dominated above 60°C and *Phormidium*-like filamentous cyanobacteria were present between 30 and 60°C. A similar importance of *Synechococcus* spp. has been found in other hot spring mats such as those in northern Thailand (pH 7.3 to 9.1, Sompong et al., 2005) or in Tibet (pH 7.0 to 8.1, Wang et al., 2013). However, in Porcelana we did not identify a significant number of *Synechococcus* spp. reads, neither did we find more than 0.5% of reads when the 16S rRNA fragments were analyzed. This confirmed our own previous studies using DGGE in Porcelana (Mackenzie et al., 2013) where presence of *Synechococcus* and *Thermosynechococcus* sequences was marginal. *Cyanobacteria* are typically absent from very acidic hot springs (i.e., Bohórquez et al., 2012), and particularly *Synechococcus* is commonly present in springs that tend to be slightly alkaline (pH 8). This might explain why this cyanobacterium was absent from Porcelana since it is slightly acidic (pH 6.5).

*Proteobacteria* were the third phylum in importance (**Figure 2A**). Their relative abundance in metagenomes increased from 5 to 30% as temperature decreased from 66 to 48°C. Interestingly, the contribution of *Proteobacteria* to metatranscriptomes was negligible at 66°C but very significant at the two lower temperatures during the night but not during the day (**Figure 2A**).

*Proteobacteria* belonged to the alpha-*Proteobacteria* (mostly *Rhodospirillales*), beta-*Proteobacteria* (mostly *Burkholderiales*), gamma-*Proteobacteria* (mostly *Legionellales*) and some delta- and epsilon-*Proteobacteria*. These data were confirmed by looking at the 16S rRNA reads retrieved from the metagenomes (**Supplementary Figure S3**). Most alpha-*Proteobacteria* reads could be assigned to the *Rhodospirillales* genus *Elioraea*, whose 16S rDNA sequences were 98.8% similar to *Elioraea tepidiphila* DSM 17972 (**Supplementary Table S3**). This bacterium was initially isolated from a hot spring at 45–50°C. More recently a second member of the genus, *Ca*. Elioraea thermophila was identified in YNP. Both of them contain genes for bacteriochlorophyll a synthesis (Tank et al., 2017). We did detect *puf*M genes from *Elioraea* sp. both in metagenomes and metatranscriptomes and this will be discussed in the next section. Our sequence was 96.7% similar to the 16S rDNA gene of *Ca*. Elioraea thermophila. Therefore, the *Elioraea* sp. from Porcelana is different from that from YNP.

Among the beta-*Proteobacteria*, a *Burkholderia* (97% similar to *Cupriavidus* sp. PIC4; **Supplementary Table S3**) was the most represented, but we also found sequences assigned to *Nitrosospira* (100% similar to *Nitrosospira* sp.; **Supplementary Table S3**).

Members belonging to phyla *Deinococcus–Thermus* and *Bacteroidetes* contributed less than 6% each to the total reads in metagenomes (**Figure 2A**). The former phylum was more abundant at 58°C, while the *Bacteroidetes* were more or less uniformly distributed. These results were consistent with the 16S rRNA reads extracted from the metagenomes, where we found 16S rDNA sequences 98.1 and 97.1% similar to *Thermus* and *Meiothermus*, respectively, among the *Deinococcus*–*Thermus*, as well as sequences 99.9% and 100% similar to *Rhodothermus* and *Sphingobacterium*, respectively, among the *Bacteroidetes* (**Supplementary Table S3**). Both phyla showed very little contribution to the reads in metatranscriptomes although we could recover some activity of the genera *Rhodothermus* and *Sphingobacterium* (both in phylum *Bacteroidetes*) in the nitrogen cycle (see below).

The remaining bacterial phyla found (i.e., *Chlorobi, Planctomycetes, Actinobacteria*, etc.) accounted for less than 1% each of the total abundance and transcriptional activity in the mat at all temperatures, both during day and night (**Supplementary Table S2**). Among these minor components of the mats, *Chlorobi* showed more transcriptional activity at 48°C during the night, while *Planctomycetes* were largely inactive at that temperature and time of the day, with most activity during the day at 66°C (**Supplementary Table S2**). The presence of *Chlorobi* has also been described previously in many hot springs (Schmid et al., 2003; Jaeschke et al., 2009; Bryant et al., 2012; Klatt et al., 2013a), although the particular members can be different in different springs. Our *Chlorobi* sequences accounted for 0.38, 0.07, and 1.96% of all the metagenomic reads at 48, 58, and 66°C, respectively. Most of them showed between 96 and 100% (average 99.3%) similarity to sequences in the genome of the phototroph *Candidatus* Thermochlorobacteriaceae bacterium GBChlB (**Supplementary Table S3**), as the closest relative. In order to confirm that most sequences belonged to *Ca.* Thermochlorobacteriaceae bacterium GBChlB we did an additional search: metagenomically retrieved 16S rRNA and *fmo*A were compared to those of *Ca.* Thermochlorobacteriaceae bacterium GBChlB and were found to be 98.9% and 94.3% similar, respectively (at 48°C and 66°C, with no signal at 58°C) confirming the identity of our sequences.

Recently *Ca*. Thermochlorobacter aerophilum was identified in YNP (Liu et al., 2012b). We checked whether this interesting organism was present in Porcelana. When looking for potential similarity to the *Ca*. Thermochlorobacter sequence, only 10 sequences showed a similarity close to 95%, but they had higher similarity to *Chlorobium*. We also used the genome of *Ca*. Thermochlorobacter to recruit sequences from the metagenomes. Only between 100 and 300 reads were retrieved from each metagenome with similarities above 80%, and all of them showed higher similarities to *Chlorobium*. Therefore, we think it safe to state that the sequence that we recovered was representative of another genus than *Ca*. Thermochlorobacter, which was either absent or present at concentrations undetectable with our sequencing depth. It is interesting to remark that both *Ca.* Thermochlorobacteriaceae bacterium GBChlB and *Ca*. Thermochlorobacter aerophilum seem to be aerobic and unable to oxidize sulfur (Stamps et al., 2014) unlike the previously known phototrophic *Chlorobi*. The organism found in Porcelana would likely belong to the *Thermochlorobacteriaceae* family.

Among the *Planctomycetes* we detected the presence of sequences 96.6% similar in 16S rDNA to *Ca*. Scalindua marina (**Supplementary Table S3**). *Ca*. Scalindua brodae has been reported as an anaerobic ammonium oxidizing bacterium (Annamox) (Schmid et al., 2003), and its presence up to 52°C has been reported in other springs such as those in California and Nevada, where anammox bacteria such as *Ca*. Brocadia fulgida, *Ca*. Brocadia anammoxidans, and *Ca*. Kuenenia stuttgartiensis have been found (Jaeschke et al., 2009). Therefore, the anammox process might be present in the Porcelana mat, but more direct evidence is necessary (see section on nitrogen below).

Archaea are an important group in some hot springs participating in methanogenesis (Merkel et al., 2015), ammonia oxidation (Dodsworth et al., 2011b; Chen et al., 2016), denitrification, DNRA (Dodsworth et al., 2011a), and other major biochemical processes (Offre et al., 2013). Archaea present in Porcelana showed transcriptional activity in the microbial mat (**Supplementary Table S2**), but they reached on average only 1.9% and 0.4% of the reads at day and night, respectively. The low representation of this domain could be due to the relatively low temperatures registered in Porcelana with respect to other hot springs, since high abundance and activity of Archaea have been only reported from 70 to 95°C (de la Torre et al., 2008; Reigstad et al., 2008; Hamilton et al., 2014). However, the presence of the *amo*A gene attributed to this group in metatranscriptomes suggests that they may have an important role as ammonia oxidizers. Specifically, *Thaumarchaeota* (*Nitrososphaera* sp.) were the most active Archaea at the three temperatures at day and night (**Figure 2B**). At the upper temperature of 66°C, *Thaumarchaeota* and *Euryarchaeota* showed similar abundances, but most of the diurnal transcriptional activity was still due to *Thaumarchaeota*. During the night, members of *Thaumarchaeota* were the only active archaeal representatives at 58 and 48°C. We found 16S rDNA sequences in metagenomes assigned to *Nitrososphaera* (16S rDNA 99.7% similar to Crenarchaeote enrichment culture clone OREC-B1045) and *Nitrosopumilus* (99.7% similar to *Nitrosopumilus* sp. DDS1) (**Supplementary Table S3**).

### Phototrophic Activity

**FIGURE 3:**
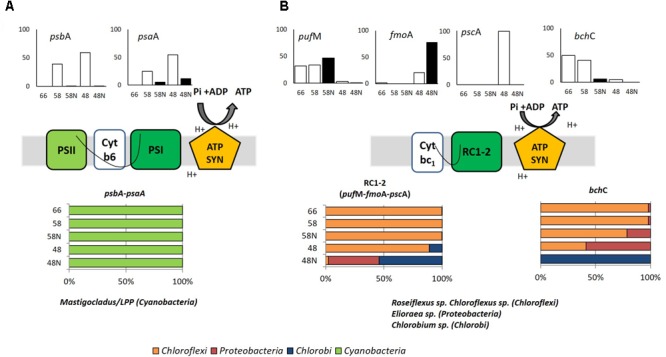
Phototrophy in Porcelana mat. **(A)** Oxygenic (*psb*A and *psa*A genes) and **(B)** Anoxygenic (*puf*M, *fmo*A, *psc*A, and *bch*C genes) photosynthesis. Cartoons in the middle part show the selected photosynthetic genes. Bar graphs show the percent of the total transcripts of each gene found in each sample, during the day (white bars) and night (black bars) at each temperature. The lower horizontal bars show the percent contribution of the main taxa to transcription for each process at each temperature. The genes considered are shown above the bars.

Several genes essential for light utilization were chosen as representative of photosystems I and II (*psaA* and *psbA*, respectively), reaction centers (RC) 1 and 2 (*pscA* and *pufM*, respectively), a bacteriochlorophyll *a* binding protein found in phototrophs that contain RC type-1 and chlorosomes (*fmoA*), and bacteriochlorophyllide *a* synthesis (*bchC*) (**Supplementary Table S4**). The two first genes are associated with the oxygenic photosynthesis carried out by *Cyanobacteria*, while the latter four are involved in anoxygenic photosynthesis or just phototrophy carried out by a variety of bacteria, including *Chloroflexi* and *Proteobacteria* (with RC type-2) and *Chlorobi* and *Heliobacterium* (with RC type-1). The transcriptional activity of these genes is shown in **Figure 3** and **Supplementary Figure S4**. **Figure 3** provides a synthetic picture, while actual transcripts for each process are presented in **Supplementary Figure S4**. Marker genes for both photosystems I and II were mostly expressed at the lower temperatures of 58 and 48°C, essentially during daytime (**Figure 3A**), with *Mastigocladus* sp. being the genus responsible for most of these phototrophic activity, as expected given its previously reported optimal growth temperature within this range (Ionescu et al., 2010; Alcamán et al., 2017). In addition to *Mastigocladus*, a minor contribution of *Oscillatoriales* of the LPP group (such as *Leptolyngbya* sp.) was detected at 66°C. These genes showed very little transcriptional activity at 66°C during the day (**Figure 3A**), in fact, it was almost two orders of magnitude lower than at the two lower temperatures (**Supplementary Figure S4a**). During the night, transcriptional activity was lower than during the day at the two lower temperatures (**Figure 3A**). Most transcripts were assigned to *Mastigocladus*, an organism that was mostly active at the two lower temperatures (**Supplementary Figure S2**). Moreover, down-regulation of photosystem and CO_2_ fixation genes in the dark has been shown for *Cyanobacteria* both in the laboratory (Hernández-Prieto et al., 2016) and in the field (Ottesen et al., 2014). At temperatures higher that 58°C, a diel study in Mushroom Spring effluent channel (60–64°C) by Steunou et al. (2008) attributed transcripts of *psb*B gene exclusively to *Synechococcus*. And in a more detailed study of phototrophy in the same spring, Liu et al. (2011) observed again that *Synechococcus* was the most important organism carrying out oxygenic photosynthesis using chlorophyll *a*.

In Porcelana, some of the transcripts found were also assigned to anoxygenic phototrophy (**Figure 3B**). These genes showed very low transcriptional activity compared to those of photosystems I and II, between one and two orders of magnitude lower (compare **Supplementary Figures S4a,b**). Most of this activity could be attributed to *Chloroflexus*/*Roseiflexus* at the two higher temperatures, while *Elioraea* sp. and *Ca.* Thermochlorobacteriaceae contributed significantly at 48°C, especially during the night (**Figure 3B**). The *bch*C gene was transcribed at the two upper temperatures during the day and essentially all this activity was due to *Chloroflexi*. At 48°C there was ten times lower activity and it was due to Chlorobi. Klatt et al. (2013b) showed that *Chloroflexus* and *Roseiflexus* expressed *pufM* and *bchC* genes preferentially in the dark in YNP, which is in accordance with the results reported here at 58°C for *pufM* but not for *bchC*. In summary in our study, the anoxygenic phototrophy shows a minor activity for the whole mat compared to the full oxygenic photosynthesis (**Figure 3** and **Supplementary Figure S4**). However, it was significant at the highest temperature. It was carried out by *Chloroflexi* at 66 and 58°C. At the lower temperature *Elioraea* sp. (an alpha-*Proteobacteria* with reaction center-2; 98.8% similar to *E*. *tepidiphila* as explained above) and a *Chlorobium* sp. (99.3% similar to *Ca.* Thermochlorobacteriaceae bacterium GBChlB as mentioned above) showed some activity. Tank et al. (2017) found *Ca*. Elioraea termophilum to be a photoheterotroph in YNP. Thus, the *Elioraea* in Porcelana must be metabolically similar to the YNP strain.

Although –considering transcriptomic data– light utilization was mostly due to the oxygenic autotroph *Mastigocladus* (**Figure 3A**), a range of light harvesting strategies was present along the temperature gradient. Our study demonstrates the importance of considering several temperatures along the gradient, since light was used by different mechanisms depending on microbial composition as modulated by temperature. This will be essential to generate a complete understanding of all potential functions carried out by the whole mat ecosystem.

### Autotrophy: CO_2_ Fixation Pathways

**FIGURE 4:**
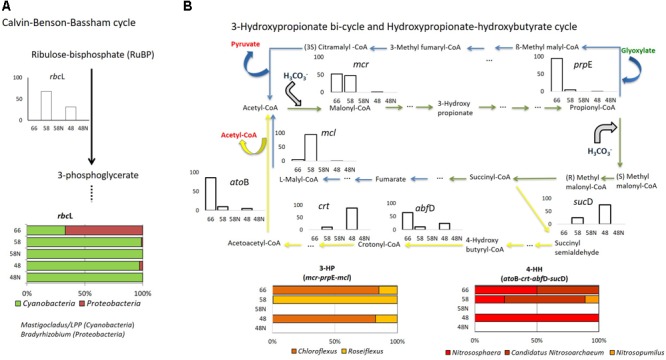
Autotrophy in Porcelana mat. **(A)** Calvin–Benson–Bassham cycle (*rbc*L gene); **(B)** 3-hydroxypropionate bi-cycle (blue arrows: *mcl, mcr*, and *prp*E genes), and hydroxypropionate-hydroxybutyrate cycle (yellow arrows: *ato*B, *crt, abf*D, *suc*D genes). Green arrows show the steps shared by the two pathways in Bacteria and Archaea. Bar graphs show the percent of the total transcripts of each gene found in each sample, during the day (white bars) and night (black bars) at each temperature. The lower horizontal bars show the percent contribution of the main taxa to transcription for each process at each temperature.

The genes chosen as representative of different carbon fixation pathways are shown in **Supplementary Table S4**. These genes were searched for in metagenomes and metatranscriptomes as in the previous section. Most of the transcription of genes involved in autotrophic CO_2_ fixation, occurred at intermediate and lower temperatures, during the day (**Figure 4A** and **Supplementary Figure S5**). The Calvin–Benson–Bassham cycle (CBB) for CO_2_ fixation, based on the large number of *rbc*L (ribulose-bisphosphate carboxylase large chain) gene transcripts (**Figure 4A**), was attributed mostly (up to 90% at the lowest temperatures) to *Mastigocladus* sp. and a small proportion to the LPP group (5%) (*Lyngbya* – *Phormidium* – *Plectonema*) at 58 and 48°C at daytime. At night, a basal signal (<0.1%) of transcriptional activity was recorded for *Cyanobacteria* (**Supplementary Figure S5**), mostly associated with *Oscillatoriophycideae* (LPP group). *Proteobacteria* (*Bradyrhizobium* sp.) had the CBB genes transcribed at 66°C (66.8%), but this was extremely low at 58°C (1.1%) and 48°C (2.8%) during the day (**Figure 4A**), without any signal at night. *Proteobacteria* were more abundant and active at the lower temperatures considering all the reads (**Figure 2**). However, the *Proteobacteria* CBB genes were more transcribed at 66°C (**Figure 4A**). Since total CBB transcriptional activity at this temperature was low (**Supplementary Figure S5**), the contribution to total CBB of *Proteobacteria* was minor. Some *Bradyrhizobium* strains like *B. japonicum* are known to be able to fix CO_2_ through the CBB (Franck et al., 2008). Some other *Bradyrhizobium* strains have the genes to synthesize bacteriochlorophyll and carry out a photoheterotrophic metabolism (Okubo et al., 2012; Hanada, 2016). Analysis of 16S rRNA fragments in metagenomes corresponding to *Bradyrhizobium* were 100% similar to *Bradyrhizobium* sp. ORS278, one of these aerobic anoxygenic phototrophs. However, it was not possible to detect the *puf*M and *bch*C genes for these organisms in the metagenomes. Therefore, the *Proteobacteria* (alpha-*Proteobacteria* such as *Bradyrhizobium*) in Porcelana show gene transcription compatible with some CO_2_ fixation activity but do not appear to have pigments to use light. It is also important to mention that transcripts related to *Proteobacteria* at 66°C, represent just ∼2% of the *Cyanobacteria* transcripts at the two lower temperatures. As already mentioned, therefore, the CO_2_ fixation associated to *Proteobacteria* was very small compared to that of *Cyanobacteria*.

The 3-hydroxypropionate bi-cycle (3-HP), characteristic of *Chloroflexi* (Hügler et al., 2002) was followed through the three key genes *mcl, mcr*, and *prpE* (**Supplementary Table S4**). These genes showed active transcription at 66 and 58°C, while at 48°C transcription was orders of magnitude lower (**Figure 4B** and **Supplementary Figure S6a**). We could not detect transcription of these genes at night. Transcripts associated with the 3-HP bi-cycle were all assigned to *Chloroflexus* sp. and *Roseiflexus* sp. (**Figure 4B**). The total number of transcripts was similar to that of the CBB cycle at 66°C but it was two and four orders of magnitude lower than the CBB at 58 and 48°C, respectively (**Supplementary Figure S5**).

Several steps involved in this cycle (noted by the green arrows in **Figure 4B**) are shared with the carbon fixation performed by Archaea through the hydroxypropionate-hydroxybutyrate (HH) cycle, while several other steps are exclusively found in Archaea (yellow arrows in **Figure 4B**). We looked for four of these genes: *atoB, crt, abfD*, and *sucD* (**Supplementary Table S4**). Transcripts of these genes were assigned to *Thaumarchaeota*, including *Nitrososphaera* sp., *Candidatus* Nitrosoarchaeum, and *Nitrosopumilus* sp., and they were found exclusively during the day (**Figure 4B**). More transcripts were found at 66°C and 48°C than at 58°C (**Supplementary Figure S6b**). However, the HH cycle only represented 0.2% of the total C-uptake pathways found in the mat (**Supplementary Figure S5**), thus accounting for a very low portion of CO_2_ fixation.

Swingley et al. (2012) found the same carbon fixation pathways at the lower temperature range (57 and 65.5°C) similar to that analyzed here (48 and 58°C) in Bison Pool (YNP). In contrast, at 67°C these authors could only detect heterotrophic metabolisms, while in Porcelana both presence and transcriptional activity of genes associated with CO_2_ fixation assigned to *Chloroflexi* and *Thaumarchaeota* were still recorded. Swingley et al. (2012) also found the reverse tricarboxylic acid (rTCA), and the acetyl-CoA cycles at higher temperatures (84 and 90°C). Thiel et al. (2017) found evidence not only of CBB and 3-HP bi-cycle, but also of the reductive TCA and Wood–Ljungdahl pathways in Mushroom Spring mat at 60°C. However, none of these previous studies presented the relative contributions of each of these processes to the ecosystem at different temperatures.

To corroborate that the transcription of genes in C-pathways found actually resulted in CO_2_ fixation, *in situ* daily rates of bicarbonate (H^13^CO_3_) assimilation were measured at 58 and 48°C, under light and dark conditions (**Supplementary Figure S7**). The high rates found on average (534 nmoles C cm^-2^ d^-1^) under light compared to (7.95 nmoles C cm^-2^ d^-1^) dark conditions, are consistent with contributions by the different CO_2_ fixation processes detected in metatranscriptomes. However, based on the number of transcripts (**Supplementary Figure S5**), most of this assimilation was referred to oxygenic photosynthesis carried out by the cyanobacterium *Mastigocladus* sp. Therefore, at lower temperatures where *Cyanobacteria* and *Chloroflexi* co-occur (Liu et al., 2011), *Chloroflexi* biomass was probably supported by its heterotrophic metabolism (Sandbeck and Ward, 1981; Anderson et al., 1987; Bateson and Ward, 1988) using by-products of cyanobacterial primary producers (van der Meer et al., 2005; Zarzycki and Fuchs, 2011; Bryant et al., 2012; Klatt et al., 2013a), although some degree of mixotrophy cannot be excluded.

Three other carbon fixation pathways are known in prokaryotes. We looked for the key genes (**Supplementary Table S4**) but the number of reads found was several orders (from 10e^-1^ to 10e^-32^) of magnitude lower than for the three cycles already described.

### Nitrogen Transformation Pathways

**FIGURE 5:**
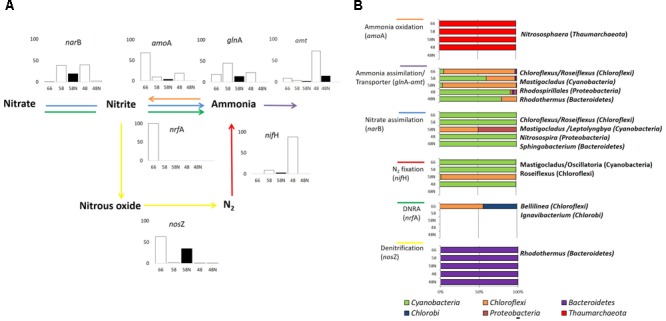
Nitrogen cycle in Porcelana mat. **(A)** Scheme of the reactions, representative genes, and transcriptional activity for each gene at the specific pathway. Bar graphs show the percent of the total transcripts of each gene found in each sample, during the day (white bars) and night (black bars) at each temperature. **(B)** Percent contribution of the main Phyla to transcription for each gene is shown by the horizontal bars. The most important genera in each case are noted to the right.

Since nitrogen is the most common limiting nutrient in Porcelana microbial mat (Alcamán et al., 2015) as well as in other circumneutral hot spring mats (Hamilton et al., 2014), pathways and genes involved in nitrogen acquisition and transformations were evaluated (**Figure 5**).

According to isotopic assimilation rates (**Supplementary Figure S7**), nitrogen fixation was the most important mechanism for the incorporation of new nitrogen to the system (Alcamán et al., 2015), and 16S rRNA and metatranscriptomics data showed that this fixation was entirely due to *Cyanobacteria* at daytime and particularly to *Mastigocladus* sp. This cyanobacterium has been recently isolated from Porcelana, and has been physiologically characterized as a thermotolerant diazotrophic cyanobacterium (Alcamán et al., 2017).

The largest numbers of *nif*H gene transcripts associated with *Mastigocladus* sp. were registered mostly at the lowest temperature (only during the day), with few transcripts at 66°C, involving sequences assigned to *Oscillatoria* sp. (96.2% similarity with *Oscillatoriales* cyanobacterium JSC-12) in addition to those of *Mastigocladus* (**Figure 5A** and **Supplementary Table S3**). The large signal of *nif*H genes correlated positively with the high nitrogen fixation rates, and also with nitrogenase activity previously reported at lower temperatures in Porcelana mat (Alcamán et al., 2015). Steunou et al. (2008) found that *nif*H activity was highest in the evening and in the early morning in Mushroom Spring. The difference with present results is due to the fact that *Synechococcus* was the abundant nitrogen fixer in YNP, and this cyanobacterium needs to separate in time oxygen production through photosynthesis and nitrogen fixation. *Mastigocladus*, on the other hand, has heterocysts and, therefore, both activities can occur at the same time.

During the night, and specifically at 58°C, *Roseiflexus* sp. expressed *nif*H gene (**Figure 5B**). It is still not clear what could be the function of the *nif*HDK-like genes in *Roseiflexus* spp. given the fact that many other accessory proteins required for the nitrogenase maturation are not present in any available *Roseiflexus* spp. genome (Thiel et al., 2017). Similarly, under controlled conditions *Roseiflexus castenholzii* is unable to grow on N_2_ as sole nitrogen source (Thiel et al., 2017).

Ammonia and nitrate assimilation rates at 58 and 48°C were three orders of magnitude lower than nitrogen fixation (**Supplementary Figure S7**). *Bacteroidetes* such as *Rhodothermus* (96.8% similarity to 16S rRNA of *Rhodothermus marinus* SG0.5JP17-172) and *Sphingobacterium* spp. (87.3% similarity with *Sphingobacterium* sp. TM-2) and *Proteobacteria* like *Rhodospirillales* and *Nitrosospira* sp. (100% similarity with *Nitrosospira* sp., **Supplementary Table S3**) also had small contributions to both processes. The large amount of *gln*A and *amt* gene transcripts, mostly associated with *Chloroflexi* at high temperatures and with *Cyanobacteria* at the lowest, indicated rather active ammonia assimilation in the mat during day and night periods. However, the number of gene transcripts of *amt* transporter were lower than those associated to *gln*A at all temperatures (**Supplementary Figure S8**). This could be tightly correlated with the very low ^15^NH_4_Cl assimilation rates recorded in Porcelana at this time (**Supplementary Figure S7**). The low natural concentrations of ammonia or nitrate measured in the system suggest that the microbial community is forced to maintain a low gene transcription rate necessary for the acquisition of these nutrients, and the preference to utilize energy in order to obtain nitrogen by N_2_ fixation.

The turnover time estimated resulted in about 1 h for ammonia. In contrast, nitrate apparently remained more time in the mat with a turnover between 12 and 34 h, evidencing that ammonia was preferred as nitrogen source in the community. High nutrient turnover rates were also found in other hot springs such as Perpetual Spring (YNP, Hamilton et al., 2014).

*Thaumarchaeota* transcripts of the *amo*A gene were detected at all temperatures, especially at 66°C (**Figure 5B** and **Supplementary Figure S8**). This indicated *Thaumarchaeota* were active as ammonia oxidizers as in other springs (Dodsworth et al., 2011b; Chen et al., 2016). This transcription was assigned particularly to *Nitrososphaera* sp. relatives (97.9% similarity with *Crenarchaeote* enrichment culture clone OREC-B1045 (**Supplementary Table S3** and **Figure 5B**). A similar pattern has been found in a biofilm growing at 63°C in a thermal artesian spring at YNP, where *Thaumarchaeota* dominated among other ammonia oxidizers (Marks et al., 2012). Other studies have also documented Archaea in hot springs with very low pH and high temperature (Hou et al., 2013), as well as at high temperatures in circumneutral springs (Cole et al., 2013). In fact, *Nitrososphaera* was the most abundant and active *Thaumarchaeota* at all temperatures, decreasing its dominance in metagenomes and metatranscriptomes with decreasing temperature. An opposite pattern was observed for other less abundant and active *Thaumarchaeota* such as *Nitrosopumilus* sp. (99.7% similarity with *Nitrosopumilus* sp. DDS1) that increased slightly with decreasing temperature. In fact, it has been reported that the thermophilic *Thaumarchaeota Nitrosophaera* sp. can grow under low ammonium concentrations such as those found in Porcelana (0.01 μmol L^-1^), with the potential to carry out CO_2_ fixation under culture conditions (Hatzenpichler et al., 2008). The widespread distribution of putative archaeal *amo* genes and their numerical dominance over their bacterial counterparts in most marine and terrestrial environments suggest that ammonia oxidizing Archaea play a major role in global nitrification (Francis et al., 2005; Zhang et al., 2008). Some studies by Dodsworth et al. (2011b) in Great Boiling Spring and Sandy’s Spring West (United States Great Basin) reported the autotrophic ammonia-oxidizing archaeon *Ca.* Nitrosocaldus yellowstonii and identified ammonia oxidation as a major source of energy fueling primary production in these extreme environments.

Finally, it was also possible to detect in the different metatranscriptomes, genes related to the principal enzymes involved in the two nitrogen dissimilatory processes: nitrate reduction to ammonium (DNRA) (*nrf*A) and denitrification (*nos*Z) (**Figure 5, Supplementary Table S4**, and **Supplementary Figure S8**). DNRA was detected only at 66°C and was taxonomically associated with *Chloroflexi* (16S rDNA 93.8% similar to *Bellilinea caldifistulae*) and *Chlorobi* (97.8% similar to *Ignavibacterium album* JCM 16511, **Supplementary Table S3** and **Figure 5B**). *Bellilinea* and *Ignavibacterium* have been previously found in hot springs. *Ignavibacterium* was isolated from Yumata hot spring (Japan), and it contains the two genes (*nrf*A as *nrf*H) as indicators of nitrite reductase (Liu et al., 2012a). The *nos*Z gene was also more actively expressed at 66°C and 58°C at night (**Figure 5A**) and was primarily attributed to heterotrophic organisms (i.e., *Rhodothermus* sp. 99.9% 16S rRNA similarity, **Supplementary Table S3**), whose denitrification activity has been shown to take place typically under low oxygen conditions (Zumft, 1997). Even though active genes of DNRA and denitrification were found at high temperatures in the mat, they both quantitatively represented very low number of reads in comparison to the other nitrogen transformation processes (**Supplementary Figure S8**).

As mentioned above, the presence of *Ca*. Scalindua (*Planctomycetes*) (16S rDNA sequence 99.8% similar, **Supplementary Table S3**) suggests the potential anammox activity of this species in the mat (Schmid et al., 2003). However, the absence of the enzyme hydrazine synthase (*hzs*A gene) in our metagenomic data and the low contribution of the *Planctomycetes* phylum (only reached <1% of reads in metatranscriptomes) at high temperatures, prevent determining the relevance of this process in Porcelana. Further studies in Porcelana mats at night when oxygen is depleted, are now needed to better understand the relevance of this and other anaerobic processes.

In summary, nitrogen fixation was the most active nitrogen uptake process at intermediate and low temperatures, and it was mostly due to *Cyanobacteria* (*Mastigocladus* sp.). At the highest and intermediate temperatures, however, ammonia oxidation and assimilation by members of *Chloroflexi* and some *Thaumarchaeota* might have a more significant contribution to the system. Probably at higher temperatures (>70°C), the input of nitrogen could be due to the contribution of others microbial members of *Cyanobacteria* by nitrogen fixation (Loiacono et al., 2012) or by Archaea through nitrification (Reigstad et al., 2008). More speculative, in Porcelana spring the nitrogen could be supplied by fumarole water inputs. Now more time-sampling points at the temperature gradient are necessary to have a more complete view of the microbial community activity inhabiting Porcelana hot spring.

## Conclusion

**FIGURE 6:**
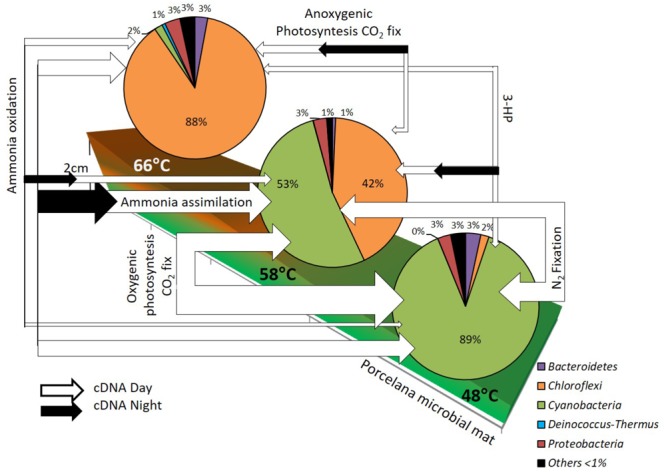
Reconstruction of the diurnal changes in active carbon and nitrogen pathways, based in metatranscriptomics analysis, along the temperature gradient in Porcelana microbial mat dominated by *Chloroflexi* and *Cyanobacteria* (pie charts). The white (day) and black (night) arrow width represent the total gene transcripts to each pathway and indicates the importance of these at each temperature.

Combining isotopic rate measurements with metagenomics and metatranscriptomics analyses allowed the reconstruction of the main carbon and nitrogen pathways, as well as light utilization forms, over a temperature gradient between 48 and 66°C in Porcelana, with the identification of main microbial players in each of these pathways (**Figure 6**). It was demonstrated that the most important taxon in Porcelana thermal mat was the cyanobacterium *Mastigocladus*, who was present at all temperatures but mostly active at the lower temperatures in the gradient. It has been shown that this cyanobacterium has an optimal temperature for growth and nitrogenase activity at 45–50°C (Alcamán et al., 2017). In culture the maximal temperature for growth was 58°C, thus, growth at 66°C is not expected. The cyanobacteria found in Porcelana at the latter temperature are likely the result of past growth when temperature distribution along the stream might have been lower. Moreover, this cyanobacterium was responsible for most of the primary production in this system through its oxygenic photosynthesis and nitrogen fixation activities.

Another relevant guild was the FAPs, represented mostly by *Roseiflexus* and *Chloroflexus*. Particularly, *Chloroflexus* can use light as a source of energy via bacteriochlorophyll *c*, carrying out anoxygenic photosynthesis according to our transcriptomics data as well as be active in CO_2_ fixation through the 3-HP bi-cycle. *Roseiflexus* might carry out the same metabolism, although no autotrophic *Roseiflexus* strain has been described so far. Since the photosynthesis genes of these bacteria are usually active during the evening or night, a role in photosynthesis at 66°C and in the other temperatures is likely. However, FAPs seem to contribute very little to the total carbon fixation in the mat although their contribution is significant at the highest temperature. On the other hand, this group was responsible for most of the ammonia uptake at the highest temperature of 66°C.

Other taxa in the mat were also noteworthy, such as the alpha-*Proteobacteria* anoxygenic phototroph *Elioraea*. This bacterium was mostly responsible for the transcription of anoxygenic phototrophic genes at 48°C during the night. In this respect, *Candidatus* Elioraea thermophila has also been found to be phototrophic in YNP hot spring mats (Thiel et al., 2016, 2017; Tank et al., 2017). Regarding ammonia oxidation to nitrate, all the *amo*A gene transcripts found were attributed to *Thaumarchaeota*, which were active at the three temperatures, likely growing chemolithoautotrophically and fixing CO_2_ via the HH cycle. Although their abundance was relatively low, and their contribution to primary production was probably minor compared to that of *Cyanobacteria*, their contribution to nitrification was likely very important in the ecosystem, particularly at the highest temperature, where there was no nitrogen fixation by *Cyanobacteria*, and nitrification could act as a new source of nitrate to the community.

One final point was the frequent decoupling between abundance and total transcriptional activity of the microorganisms at different temperatures such as those observed here for *Chloroflexi* members and *Mastigocladus*. This decoupling might be explained by changes in the temperature of the water source that would shift the distance to the source where the optimal temperature of each microorganism would be found. If temperature increased, for example, *Mastigocladus* would stop growing at that point where a large biomass would remain for a while. The cyanobacterium would then start growing actively further away from the source at lower temperatures, where biomass would be temporarily low. This emphasizes the importance of determining both abundance and activity (both measured by incorporation and fixation rates as well as transcriptional activity) along the temperature gradient to fully understand the ecology of hot spring microbial mats.

## Author Contributions

BD and CP-A designed the study. MA-A and BD did the *in situ* experiments. MA-A and CF carried out the analysis of isotope experiments. JT and DP-P did the bioinformatics analyses. MA-A, CP-A, and BD wrote the draft of manuscript. JT, CF, DP-P, and MV revised the manuscript critically and provided substantial contributions. All authors read and approved the final version of the article.

## Conflict of Interest Statement

The authors declare that the research was conducted in the absence of any commercial or financial relationships that could be construed as a potential conflict of interest.

## Footnotes

1 https://www.bioinformatics.babraham.ac.uk/projects/fastqc/

2 http://journal.embnet.org/index.php/embnetjournal/article/view/200

3 ftp://ftp.ncbi.nlm.nih.gov/genbank/

4 http://genome.jgi.doe.gov/

## Supplementary Material

FIGURE S1 Taxonomic assignment of *Chloroflexi* by metagenomic (DNA; white bars) and metatranscriptomic reads (cDNA day: gray bars; cDNA night: black bars) at the Order **(a)** and genus **(b)** levels at the three temperatures studied.

FIGURE S2 Taxonomic assignment of *Cyanobacteria* by metagenomic (DNA; white bars) and metatranscriptomic reads (cDNA day: gray bars; cDNA night: black bars) at the three temperatures studied. **(a)** Proportion of cyanobacterial reads in different Orders. **(b)** Proportion of *Stigonematales* reads in different genera.

FIGURE S3 Total reads assigned to *Proteobacteria* by 16S rRNA sequences recovered from metagenomes. *Rhodospirillales* (alphaproteobacteria) and *Burkholderiales* (betaproteobacteria) were the most abundant orders at the lowest temperature. Note that the vertical scale is logarithmic.

FIGURE S4 Total transcripts (cDNA) assigned to phototrophic processes. **(a)** Transcripts associated with photosystems PSI (*psa*A gene) and PSII (*psb*A gene) in oxygenic photosynthesis. **(b)** Transcripts (*puf*M, *fmo*A, *psc*A and *bch*C genes) associated with anoxygenic photosynthesis. Each bar represents a different temperature and daily period. Transcripts were normalized by RPKM.

FIGURE S5 Total transcripts (cDNA) associated with the autotrophic carbon fixation pathways. CC, Calvin–Benson–Basham cycle (*rbc*L gene); 3HP, 3-hydroxypropionate bi-cycle (*mcr* gene); HH, hydroxypropionate-hydroxybutyrate cycle (*ato*B gene). Transcripts were normalized by RPKM.

FIGURE S6 Total transcripts (cDNA) associated with specific carbon fixation pathways. **(a)** 3-Hydroxypropionate bi-cycle (*mcr, prp*E, and *mcl* genes) and **(b)** hydroxypropionate-hydroxybutyrate *ato*B, *crt, abf*D, *suc*D genes) cycle. Transcripts were normalized by RPKM.

FIGURE S7 Diurnal carbon and nitrogen assimilation rates recorded at 58 and 48°C in Porcelana microbial mats. Nitrogen fixation data from Alcamán et al. (2015). The black bars represent the C or N assimilation rates in the dark.

FIGURE S8 Total transcripts (cDNA) assigned to nitrogen cycle pathways: ammonia transporter (*amt* gene), nitrate assimilation (*nar*B gene), ammonia assimilation (*gln*A gene), ammonia oxidation (*amo*A gene), N_2_ fixation (*nif*H gene), DNRA (*nrf*A gene), and denitrification (*nos*Z gene). Transcripts were normalized by RPKM.

TABLE S1 Results of high throughput sequencing: raw reads, quality reads, percentage of reads corresponding to ribosomal RNA, non-ribosomal genes, non-redundant (NR) hits and percentage of the latter that could be taxonomically assigned. Numbers of sequences are shown in millions.

TABLE S2 Total percentage of Bacteria and Archaea phyla present in the entire temperature gradient of Porcelana hot spring at noon and night (N) periods.

TABLE S3 Best hits and % similarity in the SILVA database of the most relevant organisms found in Porcelana mat.

TABLE S4 Process and pathways analyzed with principal genes associated and encoding protein. All this was used as diagnostic for phototrophy, autotrophy, and nitrogen cycle transformations.
