# Supplementary figures and images for: Diurnal Changes in Active Carbon and Nitrogen Pathways Along the Temperature Gradient in Porcelana Hot Spring Microbial Mat
Source: Front Microbiol. 2018 Oct 2;9:2353. doi: 10.3389/fmicb.2018.02353 (PMC6176055; doi:10.3389/fmicb.2018.02353)

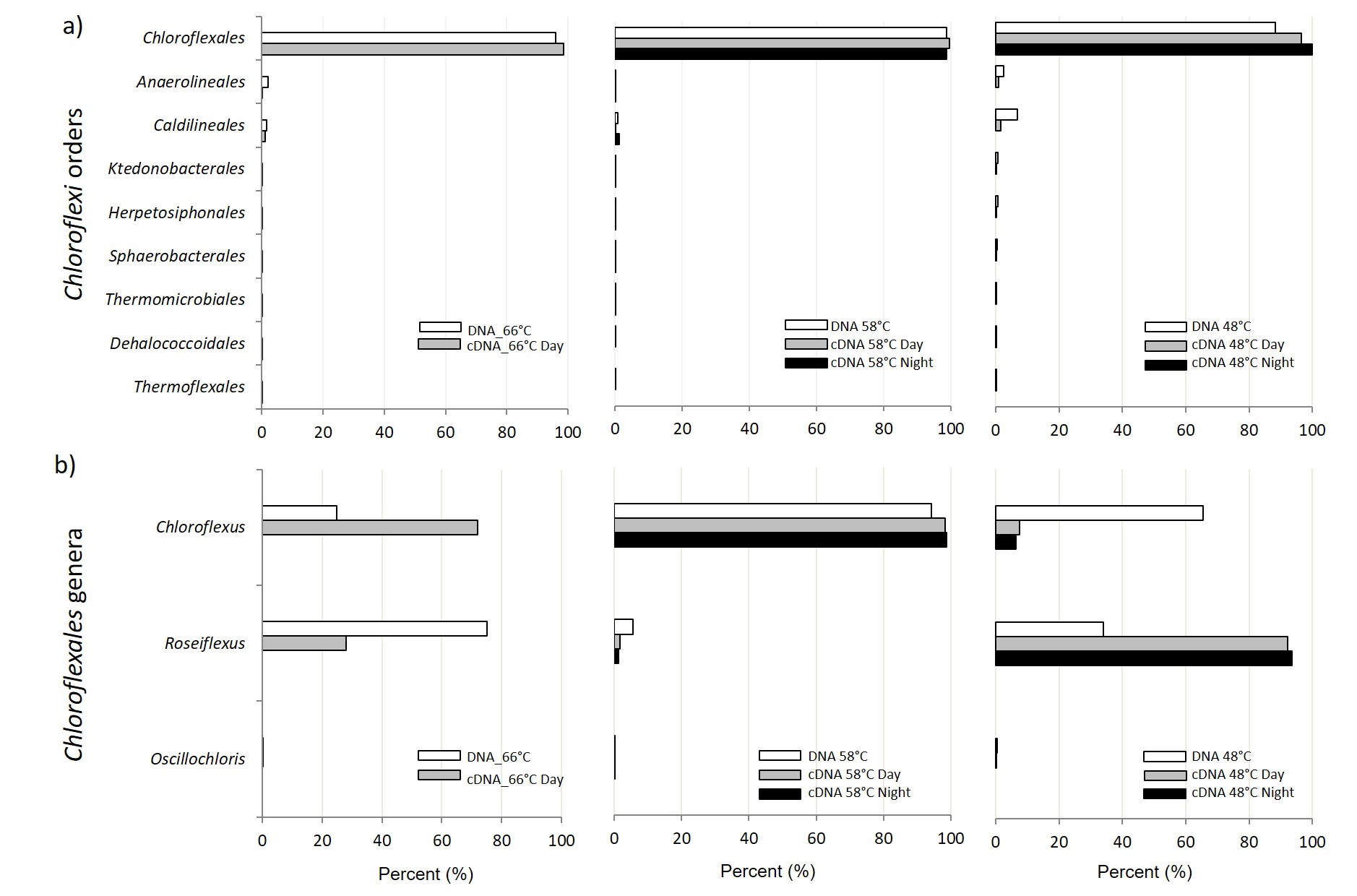

Supplement: FIGURE S1 — Taxonomic assignment of Chloroflexi by metagenomic (DNA; white bars) and metatranscriptomic reads (cDNA day: gray bars; cDNA night: black bars) at the Order (a) and genus (b) levels at the three temperatures studied. [file Image_1.JPEG]

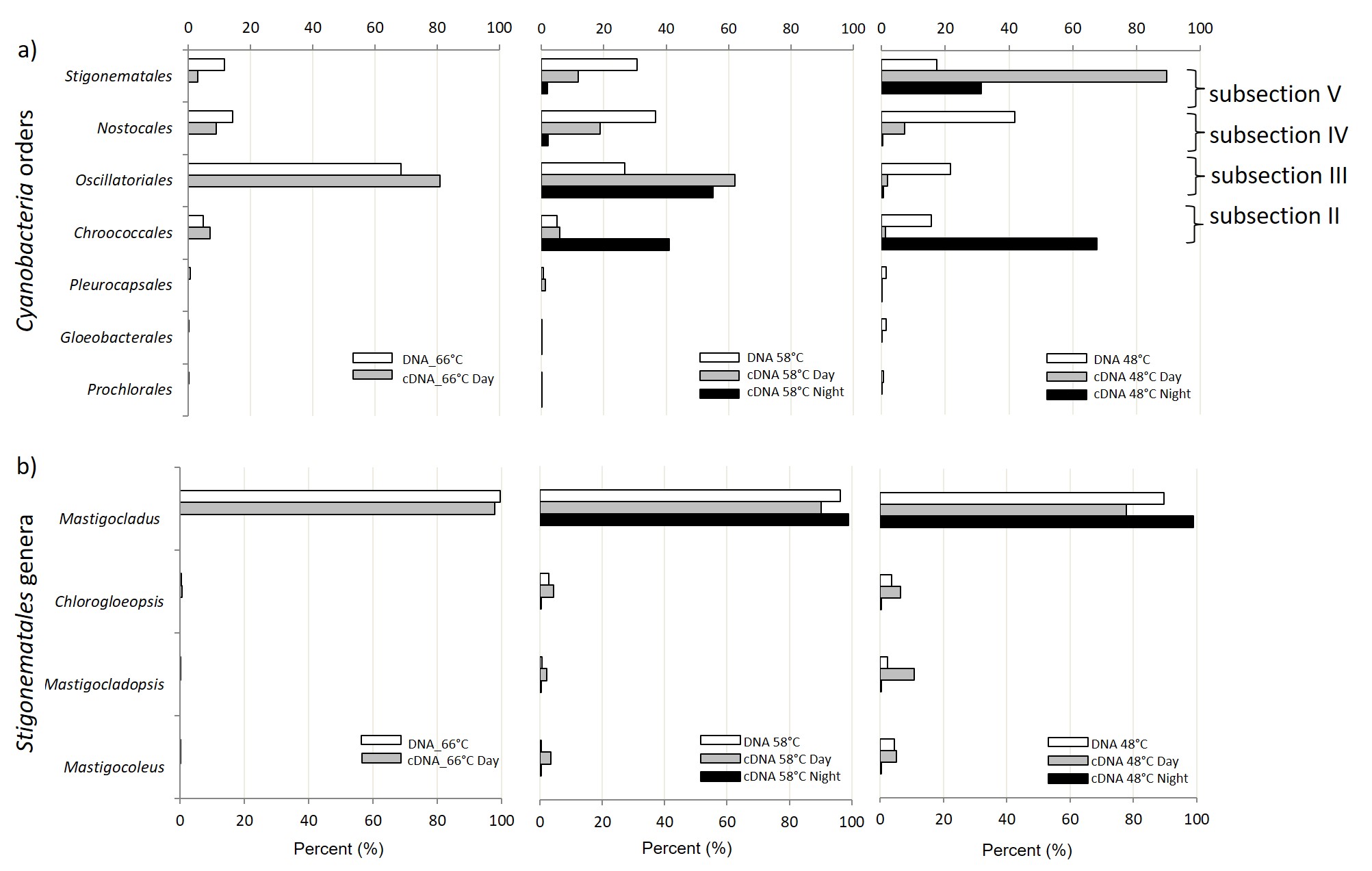

Supplement: FIGURE S2 — Taxonomic assignment of Cyanobacteria by metagenomic (DNA; white bars) and metatranscriptomic reads (cDNA day: gray bars; cDNA night: black bars) at the three temperatures studied. (a) Proportion of cyanobacterial reads in different Orders. (b) Proportion of Stigonematales reads in different genera. [file Image_2.JPEG]

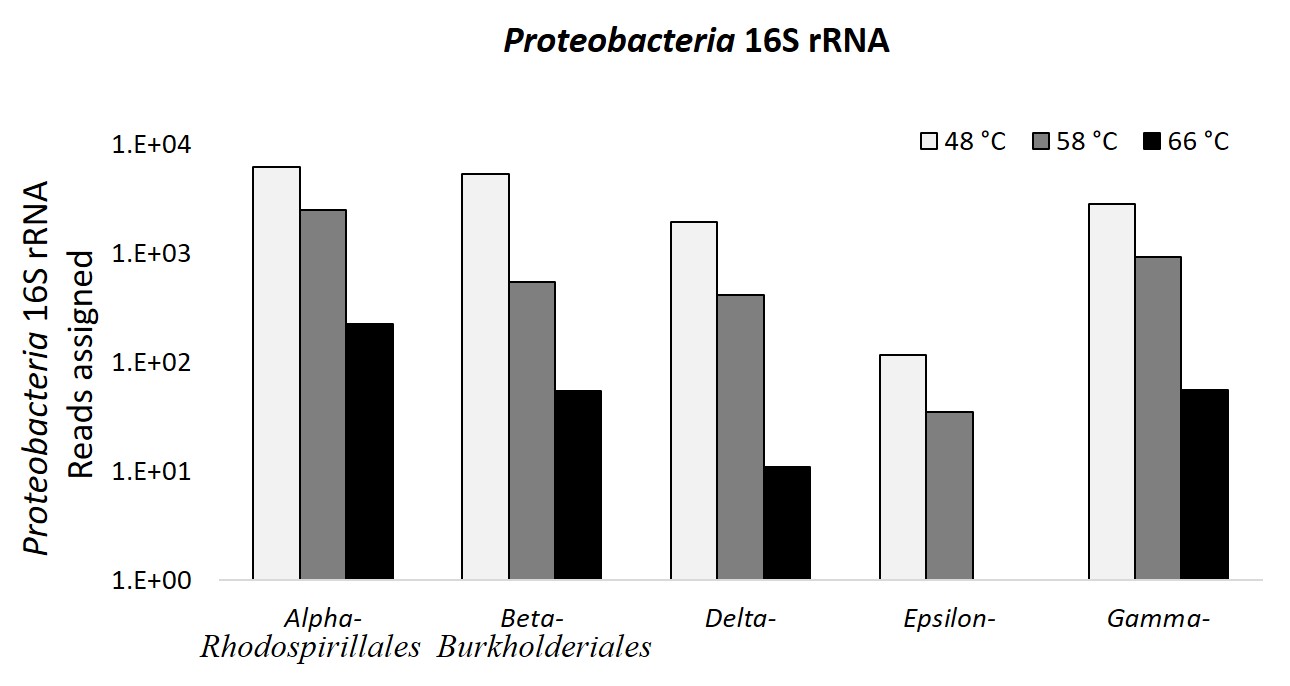

Supplement: FIGURE S3 — Total reads assigned to Proteobacteria by 16S rRNA sequences recovered from metagenomes. Rhodospirillales (alphaproteobacteria) and Burkholderiales (betaproteobacteria) were the most abundant orders at the lowest temperature. Note that the vertical scale is logarithmic. [file Image_3.JPEG]

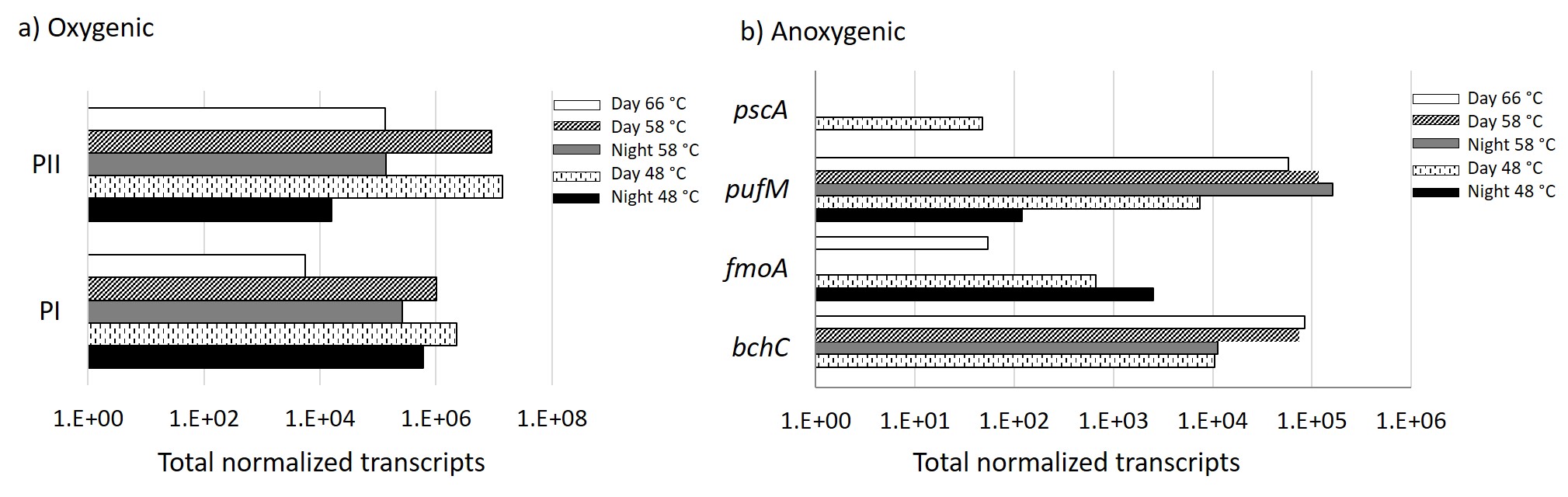

Supplement: FIGURE S4 — Total transcripts (cDNA) assigned to phototrophic processes. (a) Transcripts associated with photosystems PSI (psaA gene) and PSII (psbA gene) in oxygenic photosynthesis. (b) Transcripts (pufM, fmoA, pscA and bchC genes) associated with anoxygenic photosynthesis. Each bar represents a different temperature and daily period. Transcripts were normalized by RPKM. [file Image_4.JPEG]

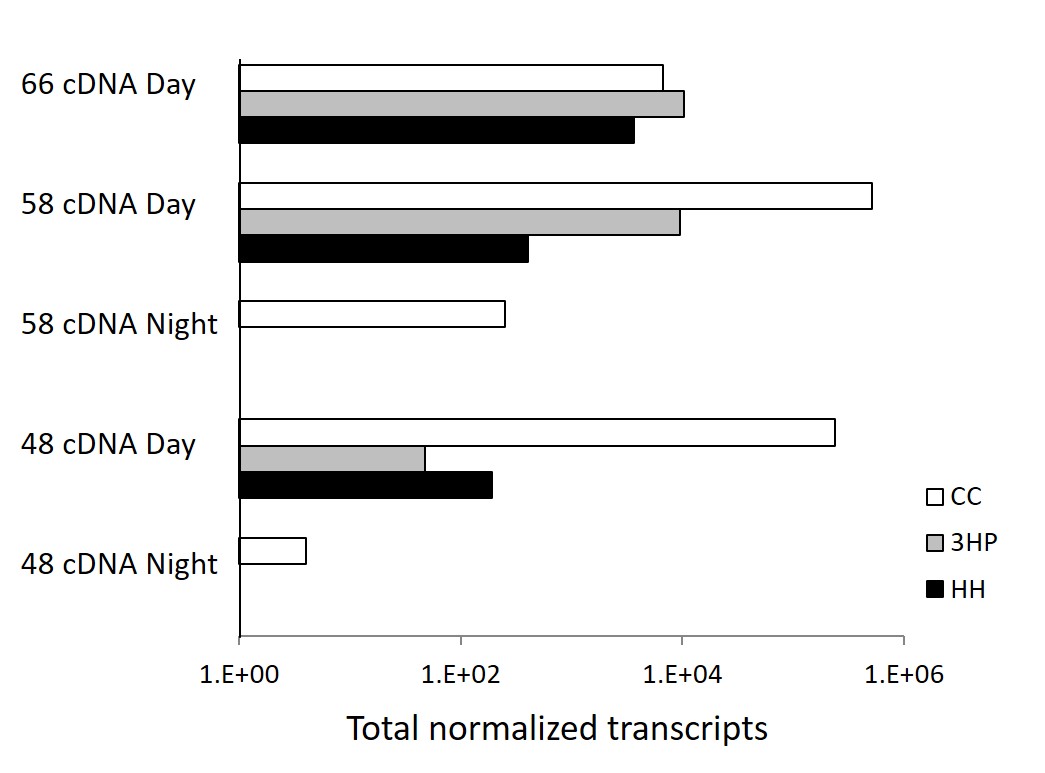

Supplement: FIGURE S5 — Total transcripts (cDNA) associated with the autotrophic carbon fixation pathways. CC, Calvin–Benson–Basham cycle (rbcL gene); 3HP, 3-hydroxypropionate bi-cycle (mcr gene); HH, hydroxypropionate-hydroxybutyrate cycle (atoB gene). Transcripts were normalized by RPKM. [file Image_5.JPEG]

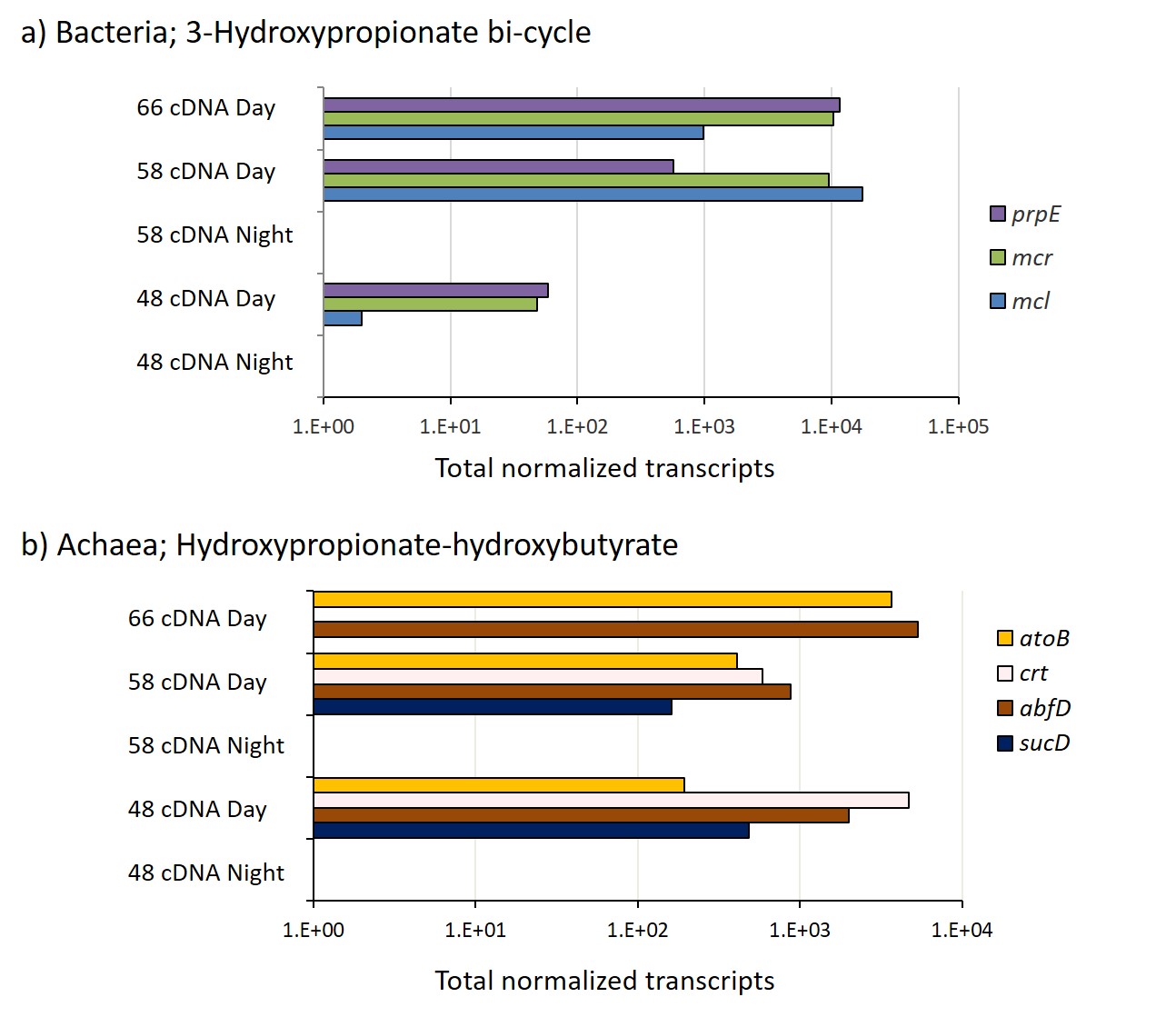

Supplement: FIGURE S6 — Total transcripts (cDNA) associated with specific carbon fixation pathways. (a) 3-Hydroxypropionate bi-cycle (mcr, prpE, and mcl genes) and (b) hydroxypropionate-hydroxybutyrate atoB, crt, abfD, sucD genes) cycle. Transcripts were normalized by RPKM. [file Image_6.JPEG]

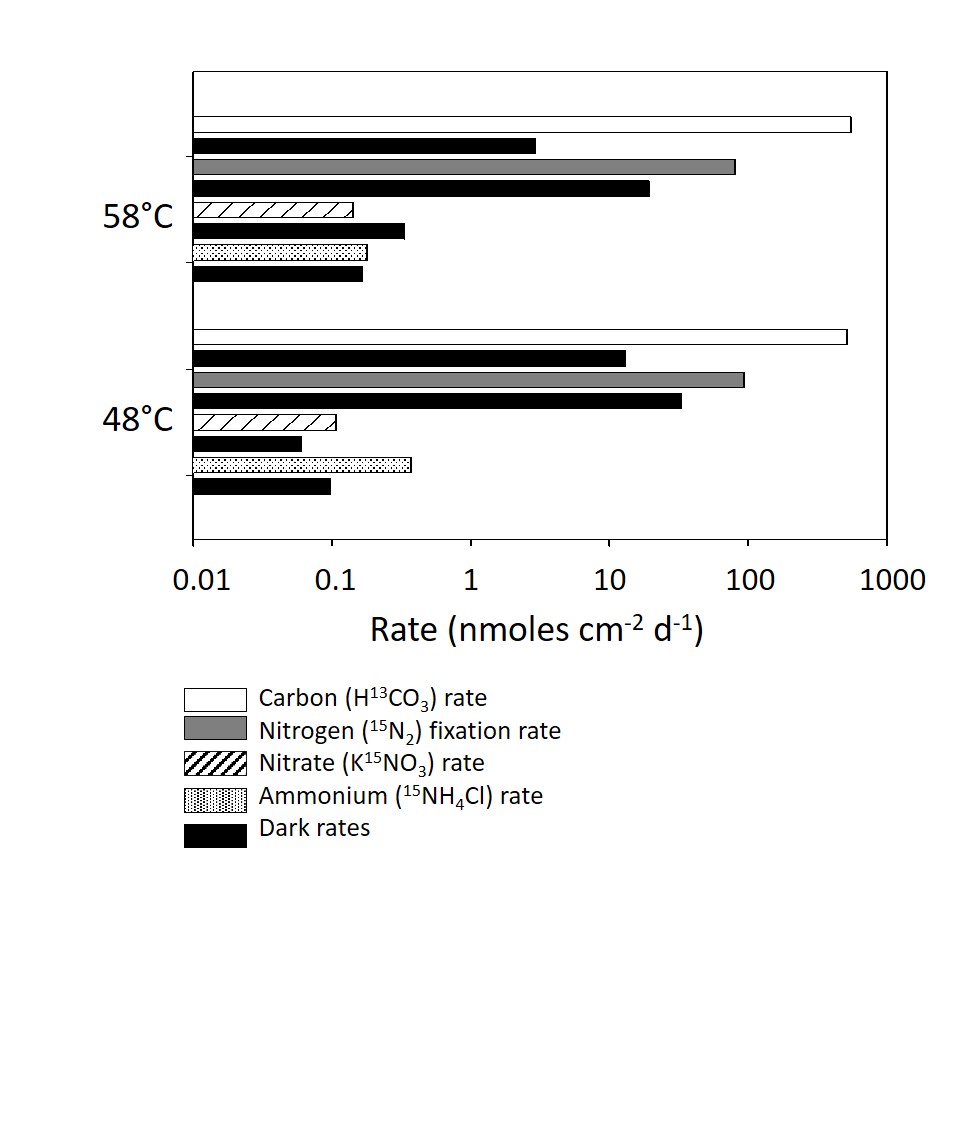

Supplement: FIGURE S7 — Diurnal carbon and nitrogen assimilation rates recorded at 58 and 48°C in Porcelana microbial mats. Nitrogen fixation data from Alcamán et al. (2015). The black bars represent the C or N assimilation rates in the dark. [file Image_7.JPEG]

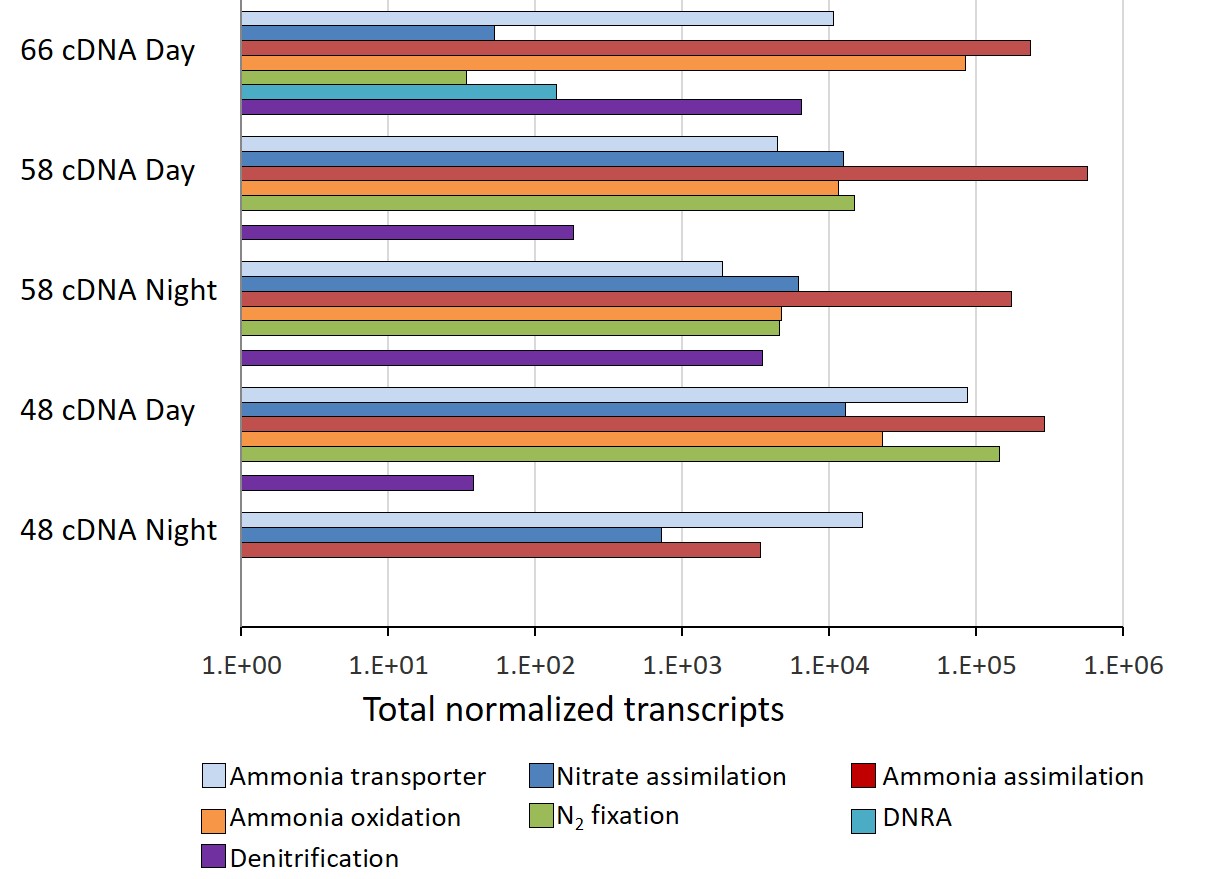

Supplement: FIGURE S8 — Total transcripts (cDNA) assigned to nitrogen cycle pathways: ammonia transporter (amt gene), nitrate assimilation (narB gene), ammonia assimilation (glnA gene), ammonia oxidation (amoA gene), N2 fixation (nifH gene), DNRA (nrfA gene), and denitrification (nosZ gene). Transcripts were normalized by RPKM. [file Image_8.JPEG]
